# Dominance of in situ produced particulate organic carbon in a subtropical reservoir inferred from carbon stable isotopes

**DOI:** 10.1038/s41598-020-69912-0

**Published:** 2020-08-06

**Authors:** Carolina de Castro Bueno, Daniele Frascareli, Erik S. J. Gontijo, Robert van Geldern, André H. Rosa, Kurt Friese, Johannes A. C. Barth

**Affiliations:** 1 Friedrich-Alexander-Universität Erlangen–Nürnberg (FAU), Department of Geography and Geosciences, GeoZentrum Nordbayern, Schlossgarten 5, 91054 Erlangen, Germany; 2 Institute of Science and Technology. Avenida Três de Março, São Paulo State University (UNESP), 511. Alto da Boa Vista, Sorocaba, São Paulo 18087-180 Brazil; 3 Department Lake Research, Helmholtz Centre for Environmental Research - UFZ, Brückstraße 3a, 39114 Magdeburg, Germany

**Keywords:** Environmental sciences, Hydrology, Limnology

## Abstract

Sources of particulate organic carbon (POC) play important roles in aqueous carbon cycling because internal production can provide labile material that can easily be turned into CO_2_. On the other hand, more recalcitrant external POC inputs can cause increased loads to sedimentary organic matter that may ultimately cause CH_4_ release. In order to differentiate sources, stable isotopes offer a useful tool. We present a study on the Itupararanga Reservoir (Brazil) where origins of POC were explored by comparing its isotope ratios (δ^13^C_POC_) to those of dissolved inorganic carbon (δ^13^C_DIC_). The δ^13^C_POC_ averaged around − 25.1‰ in near-surface waters, which indicates higher primary production inferred from a fractionation model that takes into account carbon transfer with a combined evaluation of δ^13^C_POC,_ δ^13^C_DIC_ and aqueous CO_2_. However, δ^13^C_POC_ values for water depths from 3 to 15 m decreased to − 35.6‰ and indicated different carbon sources. Accordingly, the δ^13^C_DIC_ values of the reservoir averaged around + 0.6‰ in the top 3 m of the water column. This indicates CO_2_ degassing and photosynthesis. Below this depth, DIC isotope values of as low as − 10.1‰ showed stronger influences of respiration. A fractionation model with both isotope parameters revealed that 24% of the POC in the reservoir originated from detritus outside the reservoir and 76% of it was produced internally by aqueous CO_2_ fixation.

## Introduction

Reservoirs and their tributaries are active components of landscapes. They receive, transport, process and store inorganic and organic carbon^1^. Although lakes and reservoirs cover only 2.2% of the global continental area, they may play so far poorly accounted roles in cycling continental carbon^2–4^. In addition, regional investigations of carbon budgets become increasingly important for managing water resources because they define ecosystem functions and services^5–8^.

Spatiotemporal changes of physicochemical and biological parameters in subtropical reservoirs, can increase our knowledge of carbon turnover in water bodies. For instance, excessive precipitation, can increase terrestrial nutrients, contaminants, soil leaching and additions of untreated sewage input^9^. Rainfall events can also cause near-surface turbulences in lakes that has been shown to enhance gas exchange by increasing transfer velocities of CO_2_^10,11^. Precipitation can also lead to increased input from external sources (i.e. allochthonous matter for instance from plant debris or soils) that may become trapped in lakes and reservoirs. This can, in turn, change biogeochemical dynamics of the water column^12^. Such processes include carbon uptake and release of carbon within the water column that may also be controlled by seasonal changes of temperature, weather patterns or light availability. These may in turn have important influences on carbon fluxes inside open water bodies such as for instance sedimentation, or gross primary production, ecosystem respiration and external carbon inputs^13^. For the latter, reservoir catchments may offer large but unknown contributions that may exceed internal (i.e. autochthonous) primary production^14^. On the other hand, autochthonous POC and sediment mineralization are strongly constrained^15^ and excess of this type of organic carbon can increase the emission of greenhouse gases by 20–70% (according to the mineralization rate), mainly during periods of eutrophication^16^.

Because of the important role of POC as a carbon source or sink, an essential task is to determine the origin of this carbon fraction in aquatic ecosystems. For instance, changes in terrestrial and in-lake sources of POC by photosynthesis can affect the internal organic carbon (OC) cycling in the water column^17–20^. Better knowledge of these carbon sources and sinks may also help to establish important decision tools for water quality management. This includes prevention and control of eutrophication and pollution, watershed degradation, and landscape-related processes including transportation, transformation and deposition of terrestrial material in aquatic ecosystems^21,22^.

Aquatic life relies on allochthonous carbon sources from terrestrial detritus and soils, but can also depend on autochthonous sources such as macrophytes and algae. Patterns of processing these sources of organic carbon are different. In general, allochthonous OC is more recalcitrant and has lower degradation rates and thus often becomes preferentially sequestered in sediments^23–27^. In contrast, autochthonous OC from primary production usually undergoes rapid turnover^24^. This shows the importance to understand the extents of these endmembers^28,29^. This is also reflected by the recent scientific literature that has shown increasing interest in POC dynamics, especially by means of stable isotopes of carbon fractions^21,30–33^. This demand to better understand POC dynamics also relates to the importance of integral tools to study the production and consumption of dissolved inorganic carbon (DIC) and CO_2_ through microbial decomposition, degassing, photosynthesis and respiration in aquatic systems^17,34–37^. Such approaches have hardly been applied to subtropical reservoirs (and so far nowhere in Brazil), where carbon processing may be particularly intense due to warm temperatures.

**Figure 1:**
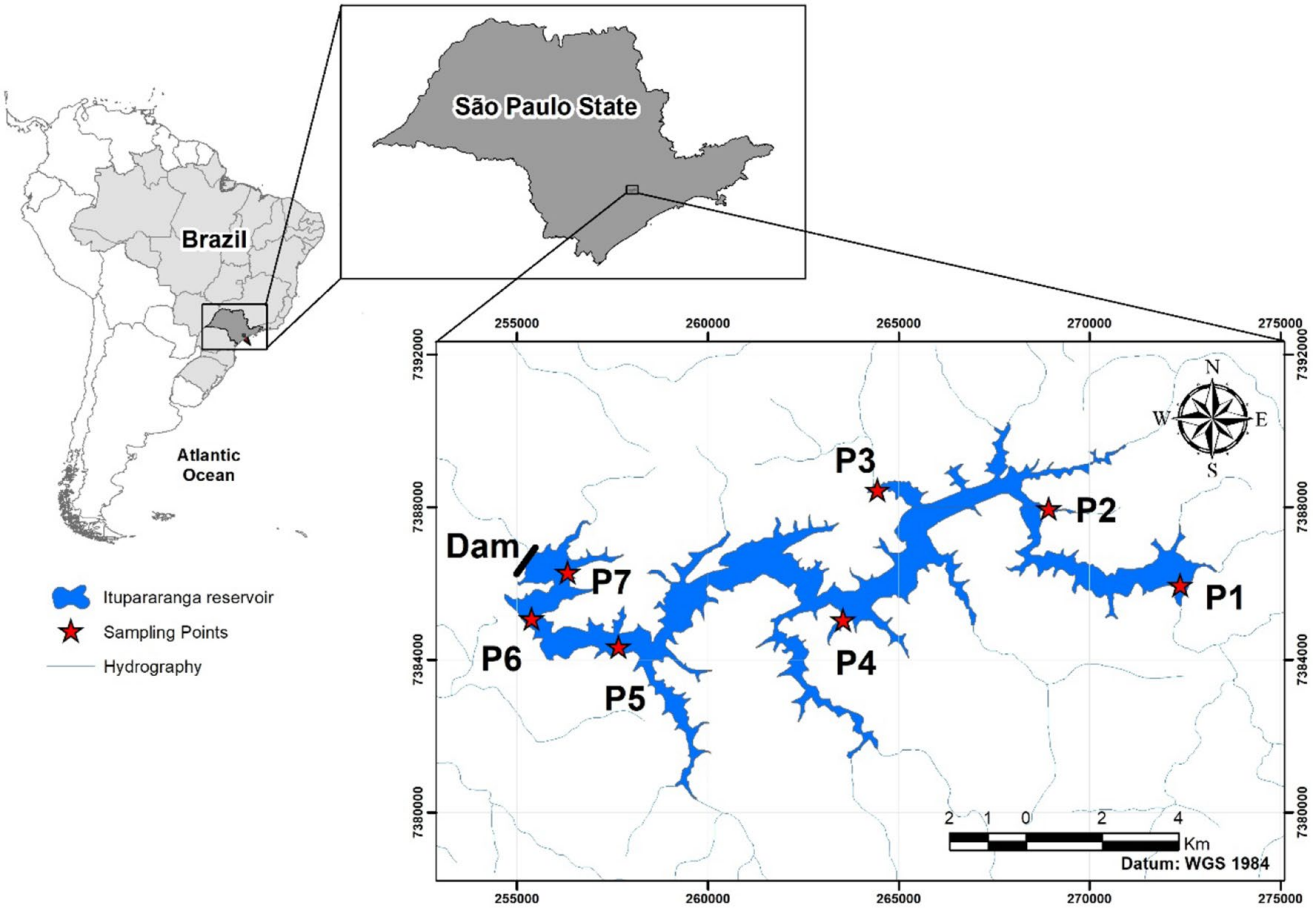
Location map of the Itupararanga Reservoir. Sampling sites P1 to P7 were sampled between December 2016 and December 2018 and are indicated by red stars. The reservoir boundaries were outlined using RapidEye images (5-m spatial resolution from 17 July and 30 August 2014) provided by Ministry of Environment and their drainage systems (1: 25.000) were downloaded from DataGeo (https://datageo.ambiente.sp.gov.br/app/#). All data processing operations were carried out using the software ArcGis 10.2.2 (ESRI, USA) and the maps were projected onto a common coordinate system (World Geodetic System 1984 [WGS 84]).

This case study was carried out in the Itupararanga Reservoir that is an important freshwater body in the state of São Paulo (Brazil) and has been monitored since 1998 by the State of São Paulo Environmental Company^38^ [https://cetesb.sp.gov.br/aguas-interiores/publicacoes-e-relatorios/—in Portuguese]. This monitoring revealed that this subtropical reservoir faces progressive deterioration in water quality over the past three decades (Fig. 1). Such processes and related carbon cycling were investigated by POC/chl-a ratios and also by combined application carbon isotope ratios (δ^13^C_POC_ and δ^13^C_DIC_) and aqueous CO_2_ contents.

This subtropical reservoir is economically and environmentally important for the metropolitan region of São Paulo. The general deterioration of environmental and water quality of the Itupararanga system has attracted the interest of several researchers^39–43^. These studies considered aspects of nutrients and cyanobacteria communities, sedimentary macroinvertebrates, metal toxicity and land-use in relation to water quality. This work has shown that the Itupararanga Reservoir receives substantial amounts of nutrients, mostly because of agricultural land use in the surrounding catchment^42^. Another important source of nutrients is sewage discharge from urban areas. They mainly reach the reservoir untreated at the entrance^39,41^. These inputs can stimulate primary production and may also cause eutrophication. This terrestrial nutrient input also suggests that organic matter (OM) and detrital material may enter the reservoir by streams and overland flow via an extensive shoreline. However, less attention has been paid to other carbon sources in this system. Therefore, the aim of this study is to advance knowledge on the reservoir-internal origin of POC by exploring spatiotemporal variations of this carbon fraction together with stable isotope ratios of DIC and POC. This also helps to outline terrestrial inputs. This technique has so far hardly been applied to the Itupararanga Reservoir that can be seen as a representative for a subtropical water body and we introduce the approach as a tool to further understand carbon cycling water systems.

## Results

**Figure 2:**
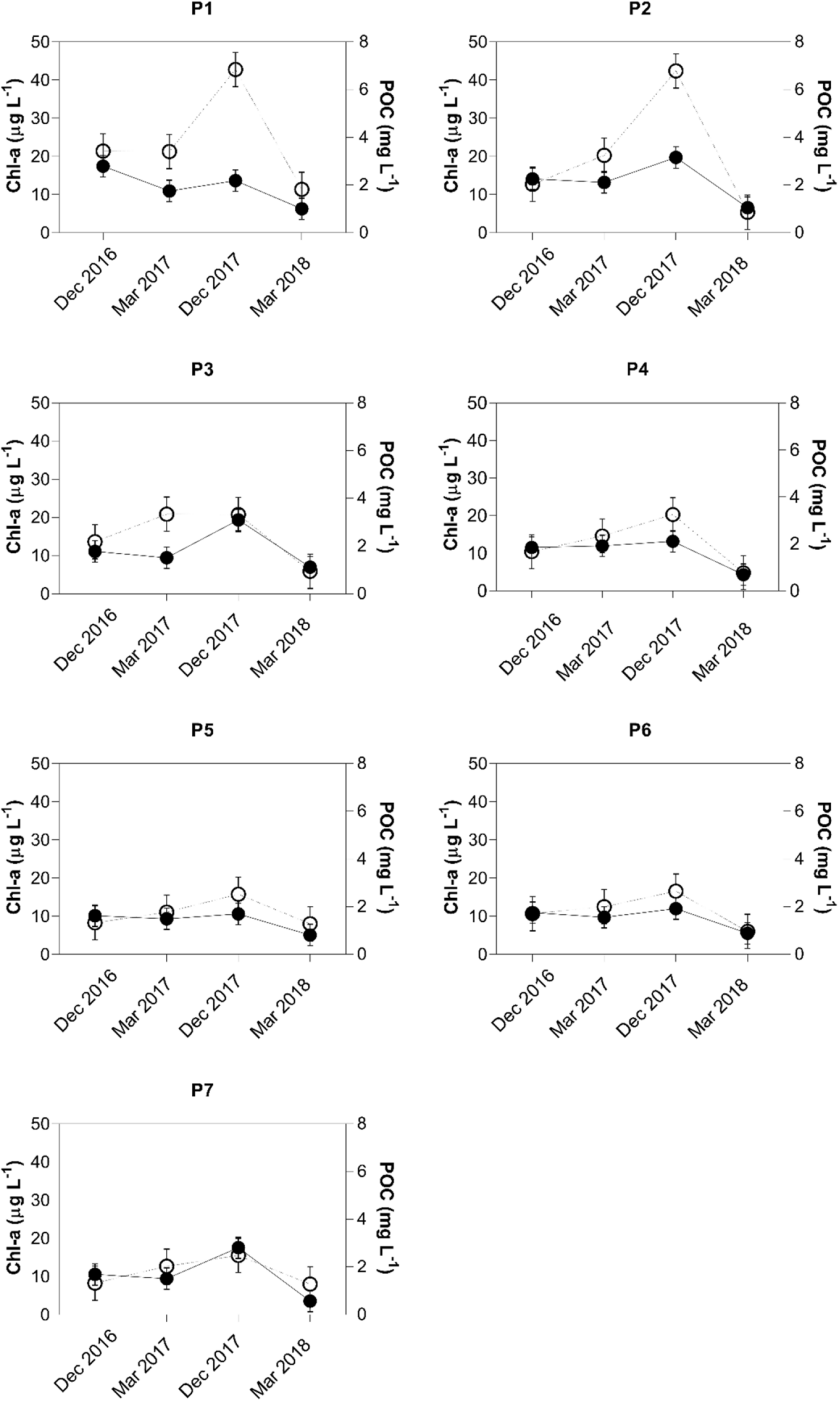
Concentration patterns of chl-a (white symbols, left axis) and POC (black symbols, right axis) at the seven sampling locations in the Itupararanga Reservoir. Samples presented in this graph were only collected in the top 3 m of the water column. Error bars represent 1σ standard deviations as determined from selected triplicate analyses.

Increased concentrations of both, POC and chl-a were observed at all surface sampling locations at depths above 3 m in December 2017, with maximum values of 2.2 mg L^−1^ and 42.8 μg L^−1^ for POC and chl-a (Fig. 2). After this season, a decrease of chl-a and POC concentrations were found in all seven sampling locations between December 2017 and March 2018. The average decrease was 67% for chl-a and 62% for POC and a maximum chl-a decrease of 87% was found at P2. The maximum decrease for POC was 80% at P7. POC and chl-a values at the seven sampling locations in the Itupararanga Reservoir are displayed in Fig. 2.

**Figure 3:**
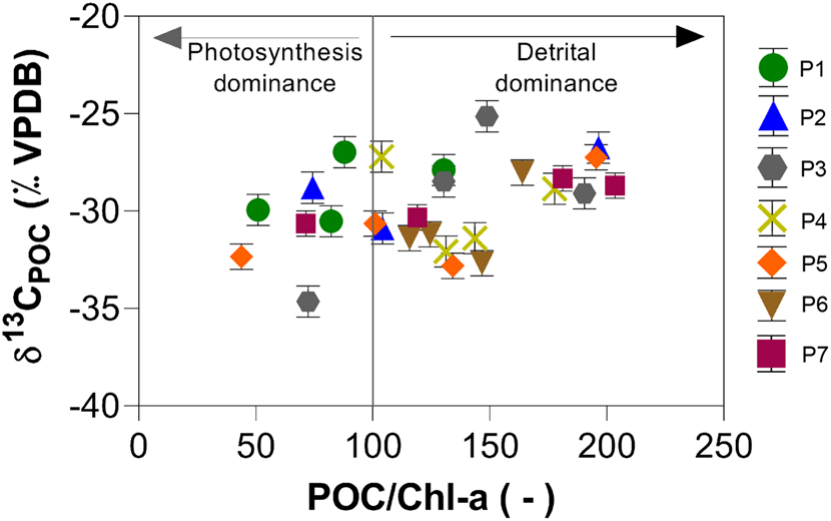
POC/chl-a ratios *versus*${\updelta }^{13} {\text{C}}_{{{\text{POC}}}}$ at the seven sampling locations from December 2016 to March 2018 in the top 3 m water depth. Symbols below the POC/chl-a ratio of 100 indicate dominance of photosynthetic sources of POC, the group with POC/chl-a ratios above 100 indicate samples with dominance of detrital sources of organic carbon. Error bars represent 1σ -standard deviations as determined from triplicate analyses of selected samples.

In a first attempt to outline sources, we plotted POC/chl-a ratios against ${\updelta }^{13} {\text{C}}_{{{\text{POC}}}}$ values (Fig. 3). Here we used a threshold of 100 for POC/chl-a ratios to define dominance of photosynthesis over detrital input^44^. According to this approach, the primary production dominates in only 26% of the surface water samples. These samples are from P1 in March 2017, December 2017 and March 2018, from P2 in December 2017, from P3 in March 2017, from P5 in December 2017 and from P7 in March 2018.

In order to test how carbon might be exchanged between DIC and POC, we investigated their stable isotope values in a Spearman correlation. It indicated positive and significant trends with r values between 0.79 and 0.98 and p values between less than 0.0001 and 0.0182 (Table 1).

**Table 1:** Spearman correlation coefficients (*r*), *p*-values (*p*) and the number of samples (n) for ${\updelta }^{13} {\text{C}}_{{{\text{POC}}}}$ versus ${\updelta }^{13} {\text{C}}_{{{\text{DIC}}}}$.

Sampling point	*n*	*r*	*p*
P1	9	0.87 ( +)	0.0066
P2	9	0.79 ( +)	0.0182
P3	10	0.87 ( +)	0.0008
P4	9	0.85 ( +)	0.0088
P5	10	0.98 ( +)	0.0001
P6	11	0.84 ( +)	0.0002
P7	21	0.79 ( +)	< 0.0001

The positive correlation is indicated by a “ + ” symbol within parentheses.

**Figure 4:**
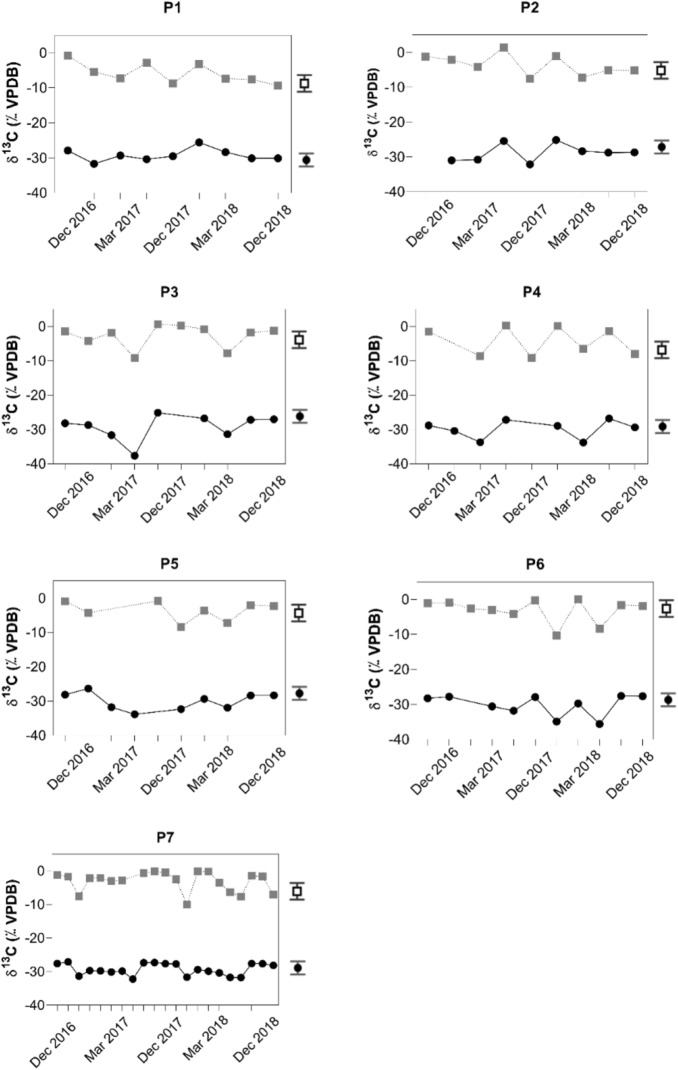
Temporal distributions of δ^13^C_DIC_ (upper curves) and δ^13^C_POC_ (lower curves) for the seven sampling locations in the Itupararanga Reservoir. Each point in the graphs refers to a sampling depth in the water column, which is shown in the Tables S1–S7 (supplementary material) The 1 − σ precisions δ^13^C_POC_ and δ^13^C_DIC_ were ± 0.3‰ and ± 0.1‰, respectively. These are shown by a symbol with error bars on the right side of the graphs.

Samples from depths below 3 m showed lower values for ${\updelta }^{13} {\text{C}}_{{{\text{DIC}}}}$ that ranged from—10.4‰ to—7.0‰ for all campaigns. Representative values from this group are—8.8‰ for P1 in December 2017 at 3 m,—7.5‰ for P2 in December 2017 at 5 m,—9.2‰ for P3 in March 2017 at 11 m,—9.2‰ for P4 in December 2017 at 8 m,—8.4‰ for P5 in December 2017 at 11 m,—10.4‰ for P6 in December 2017 at 15 m, and—10.1‰ for P7 in December 2017 at 14 m depth (Fig. 4). For the same sampling locations representative samples from the surface had more positive ${\updelta }^{13} {\text{C}}_{{{\text{DIC}}}}$ values with + 1.5‰ for P2 in December 2017 at 1 m, + 0.6‰ for P3 in December 2016 at 1 m, + 0.3‰ for P4 in December 2017 at 1 m and + 0.04‰ for P6 in March 2018 at 1 m depth (Fig. 4).

### Carbon isotope fractionation

**Figure 5:**
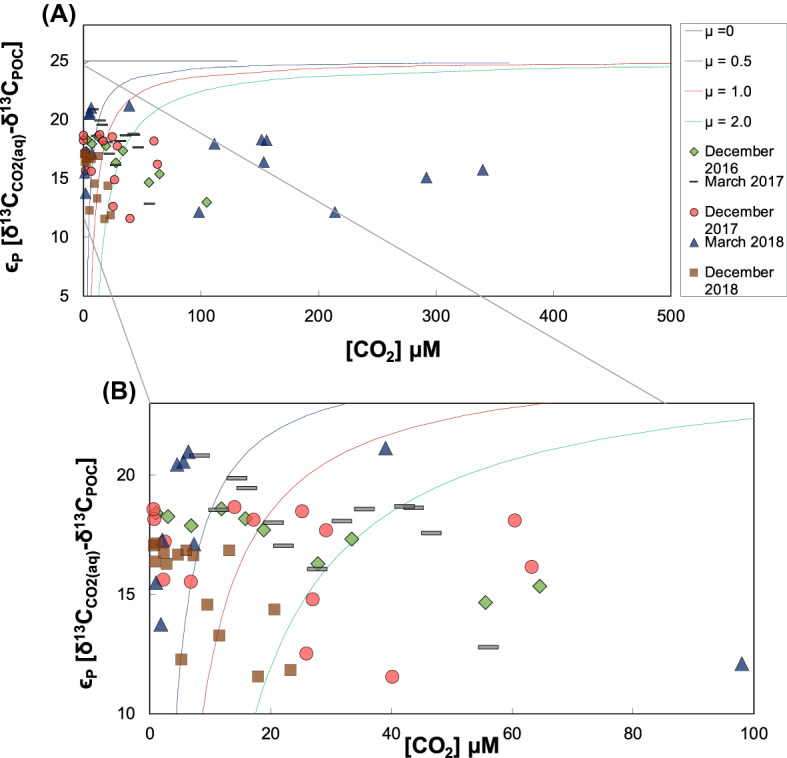
Entire set of samples compared to the model by Rau et al.^55^. (**A**) Theoretical (solid lines) and measured fractionation values (symbols) (ϵ), between CO_2(aq)_ and phytoplanktonic POC with varying growth rates (μ). (**B**) Zoom-in. Samples that plot between the curves are of autochthonous origin. Samples below the green solid line (μ = 2.0) do not follow the model by Rau et al.^55^ and this group rather indicates samples of allochthonous origin.

The data were also plotted according to a fractionation model by Rau et al.^55^. In this approach the aqueous CO_2_ contents are plotted *versus* isotope differences between ${\updelta }^{13} {\text{C}}_{{{\text{CO}}2}}$ and ${\updelta }^{13} {\text{C}}_{{{\text{POC}}}}$ according to Eqs. (2)–(5) (Fig. 5). The same was repeated for surface samples in order to afford the best comparion with the POC/chl-a ratio data (Fig. S1).

Figure 5 shows that 80% of the samples collected during the period from December 2016 to December 2018 agree with the fractionation model by primary producers. In contrast, the signal of possibly detrital POC (samples that did not fit the Rau fractionation model) appeared in the warm seasons of December 2017 and March 2018, respectively (Table S10—supplementary material). When treating only the surface samples with this approach, 95% of all samples fitted the model (Fig S1). The ones that did not agree with the fractionation model are samples from P1 in December 2017 and March 2018, both at a water depth of 3 m.

## Discussion

The possible significance of subtropical reservoirs in global and regional carbon budgets is still vague. One reason for this is the complexity and variability of external carbon inputs to these systems. Here, we investigated seasonal and spatial variations of POC and chl-a. These parameters can be combined to POC/chl-a ratios. However, this approach only offers a subjective indicator for dominance of external or internal POC sources. Combined investigation of δ^13^C_POC_ and δ^13^C_DIC_ values seem more promising to help separating POC sources, because this technique also maps transformations of carbon. Therefore, the application of a carbon stable isotope fractionation model should be able to provide distinct information on POC sources.

When looking at concentrations of POC and chl-a, most samples in Fig. 2 showed similar spatio-temporal distributions of both parameters. Note that P1 showed no considerable changes in chl-a in December 2016 and March 2017 (i.e_._ the warm season with precipitation of 148 and 146 mm, respectively). This observation was made even though the corresponding POC concentrations decreased. However, March 2017 and December 2017 had similar precipitation patterns with averages of 148 and 157 mm, respectively, but both chl-a and POC increased. Despite similarities of rainfall averages in both sampling periods, individual intense events may have increased the flushing of nutrients into the reservoir. This may in turn have caused POC generation by primary production. These trends agree with the findings of other studies^44–46^.

Sampling locations P2, P3, P5, P6 and P7 showed decreasing trends in POC from December 2016 to March 2017. These were not matched by increasing chl-a concentrations until the end of December 2017. This trend might be related to declining inputs of detrital material, organic matter consumption, or sedimentary deposition. On the other hand, increases in chl-a contents in the same period was likely caused by supply of phytoplanktonic POC. This was confirmed by an observed algae bloom in the same period, according to the Inland Water Quality Report, produced by CETESB (São Paulo State Environmental Company) in 2017. Higher concentrations of both parameters shown in Fig. 2 may also reflect algae blooms in the reservoir in December 2017.

Low POC/chl-a values in Fig. 3 indicated a dominance of photosynthesis (phytoplankton) in the POC pool. Similar observations were also made by other authors in other open aqueous systems^44,47^. Even though it is not possible to detect clear seasonal and spatial patterns from this approach, more than half of the samples were classified with origin of detrital dominance (Fig. 3). The other group of samples belong to the warm periods of March 2017, December 2017, and March 2018. They all seem to be characterised by a dominance of photosynthesis. Nevertheless, some sample points of these sampling events also plotted in the detrital field of Fig. 3.

Even though all samples for the chl-a measurements belong to the surface ($\le$ 3 m depth), their POC/chl-a ratios suggest that 74% of the POC in the Itupararanga Reservoir originate from detritus outside the reservoir. One explanation is that the POC/chl-a threshold ratio of 100 may only serve as a rough and arbitrary indicator for algae versus detrital carbon input. Moreover, this threshold value may vary over time and space. Overall, one would expect much more samples to plot in the field of photosynthesis when considering the known nutrient inputs to the reservoir and the observed algae blooms. One intermediate conclusion is that the POC pool is more complex and cannot be explained with chl-a and POC concentration data alone. This would be plausible, because primary production in complex communities impact aquatic environments via numerous metabolic interactions^48^.

The Spearman correlation in Table 1 indicates strong relationships between ${\updelta }^{13} {\text{C}}_{{{\text{POC}}}}$ and ${\updelta }^{13} {\text{C}}_{{{\text{DIC}}}}$. This is a good indicator for the possibility that both variables may have been affected by similar processes with biological productivity being most plausible. This means that processes affecting DIC isotope fractionation result from transformation of this carbon source via organic carbon production (photosynthesis) and utilization (respiration).

More ^12^C-enriched values of ${\updelta }^{13} {\text{C}}_{{{\text{DIC}}}}$ ranged from—10.4‰ to—7.0‰ and suggest respiratory signals that were most obvious at sampling location P1 (Fig. 4). Such ^12^C-enriched values of ${\updelta }^{13} {\text{C}}_{{{\text{DIC}}}}$ have also been observed in other studies^49,50^. The fact that these values were observed only for samples from water depths of three meters and below is plausible, because beyond this depth limited penetration of light hampers photosynthesis. This process usually enriches the remaining DIC in ^13^C. Additionally, increased rainfall may also have flushed ^13^C-depleted DIC and POC from the surrounding catchment^17^. The observed more positive δ^13^C_DIC_ values near the water surface likely result from equilibration with atmospheric CO_2_ and photosynthesis that both enrich the remaining DIC in ^13^C.

For a more detailed investigation, we plotted the data according to a model described by Rau et al.^55^. Overall, the majority of the samples collected between December 2016 and December 2018 revealed a dominance of reservoir-internally produced POC. This may indicate a typical pattern for tropical and subtropical reservoirs with higher temperatures, critical nutrient inputs, and generally enhanced biological activities. The sample group that conforms to the model by Rau et al.^55^ covers all sampling depths across all seasons. This is a good indication that POC was predominantly produced autochthonously with low spatiotemporal variability. When photosynthetic POC was found outside the photic zone, it likely was produced near the surface and afterwards moved downwards through the water column. The samples that do not fit this model (Fig. 5B), indicate a dominance of allochthonous POC that do not follow the same carbon transfer patterns between aquatic CO_2_ and POC. This confirms other studies that also showed allochthonous inputs to produce isotope differences between POC and DIC (ε) that are below the modelled lines by Rau et al.^44,51^.

For a better comparison to the alternative method of POC/chl-a rations, we only considered samples from within 3 m water depth in the Rau Model (Fig. S1 in supplementary material). This shows that only 5% of the sub-data set did not agree with the model (i.e. P1 in December 2017 and P1 in March 2018, both at 3 m depth). The agreement with the model of the rest of the surface water samples in the photosynthetically active zone is a good indicator of biological in situ POC generation by photosynthesis. This model interpretation contrasts with results of POC/chl-a ratios (Fig. 3), that revealed different contributions for autochthonous and allochthonous sources of carbon with 26 and 74%, respectively. This difference is surprising, because the samples for chl-a were also collected in the photic zone of the top 3 m of the water column. One reason for this discrepancy may be that more detrital POC occurs in the reservoir after strong rainfall and storms. These could also mobilize chl-a from the landscape from plant residues. In such a process POC/chl-a ratios could be different to those of freshly produced algae material and mask the effects of in situ photosynthesis. Similar masking effects might exist after re-suspension of sedimentary POC. However, the arbitrary definition of 100 of POC/chl-a ratios for the separation of photosynthesis and detrital is likely the strongest reason for the disagreement between both approaches. For instance, setting this threshold to 150 would result in about half of the samples resulting from terrestrial input.

The fact that many data fit the model at different specific growth rates (μ) may also indicate a seasonal sequence of primary producer types such as *Bacillariophyceae*, *Achnanthidium minutissimum*, *F. delicatissima var. delicatissima*. Such varieties of different algae were also observed in previous studies^52,53^. This observation is also supported by seasonal patterns of δ^13^C_DIC_ and δ^13^C_POC_ in the data set. Because the investigated seasonality intervals span several months, autochthonous POC can continually be produced in surfaces water and likely has more persistent effects on the reservoir ecosystem than terrestrial POC^34^.

Overall, simultaneous considerations of δ^13^C_DIC_ and δ^13^C_POC_ together with the availability of CO_2(aq)_ in the Rau Model, marks the transfer of carbon from the DIC to the POC phase. It should therefore be a better indicator of biological use of carbon in the form of algae growth. It also shows how carbon stable isotope fractionation depends on rate-limiting sources and cell size. Thus, this approach seems better in capturing complexities of carbon transfer by algae growth^54^. Nonetheless, the Rau Model can only predict assimilation of aqueous CO_2_ and does not account for direct uptake of aqueous HCO_3_^-^ from the water column. This may ignore a significant part of an algae community that operates in this way^55^. Moreover, at sites close to the shore, active macrophytes may alter the results by influencing the δ^13^C_DIC_ that also serves as an input parameter to the Rau Model. Even if macrophytes contribute to the POC pool by plant fragments, they may follow different isotope enrichment patterns that were not investigated here.

The fractionation model by Rau et al.^55^ was tested for all data and all periods of sampling. It successfully narrowed down the complexity of carbon assimilation to specific fractionation, growth-rates (μ) and extracellular CO_2(aq)_ concentrations. Because it revealed that the majority of samples from less than 3 m water depth (i.e. the photic zone) agreed with this model, photosynthesis and CO_2(aq)_ consumption seems to be the dominating process for POC production. Also, for some samples collected at deeper water depths than 3 m, the model produced plausible results, thus indicating vertical transport of biologically produced POC in the water column.

When considering all data points of this study, about twenty percent of POC samples did not fit the model and plotted below the theoretical lines. These samples most likely correspond to detrital input or re-suspension of sedimentary matter. The latter may either originate from upwelling during storms that in addition can also import more POC from outside the reservoir. These findings can also extend to other freshwater systems and therefore offer a tool to separate POC input pathways that in turn may have important controls on cycling of CO_2(aq)_. This would for instance be the case, if more autochthonous POC is present. On the other hand, predominant POC from terrestrial sources may imply more generation of methane from sediments. This is because more recalcitrant allochthonous material is usually less readily consumed in the water column and becomes more likely deposited in sediments. Here anoxic conditions may produce methane over longer time periods than residence times in the water column.

Overall, our results suggest that stable isotopes can help to trace sources of POC. Such combined applications of stable carbon isotope and physicochemical parameters are promising to provide sensitive differentiations of carbon sources in natural waters.

## Methods

### Study site

The Itupararanga Reservoir is located in a subtropical climate that belongs to the Cwb-type, according to Köppen classification^56^. It is situated in the upper Sorocaba River catchment in the state of São Paulo, Brazil (Fig. 1). The climate is characterised by a dry season from April to September and a wet season from October to March. The monthly average temperature is above 18° C for all months, and reaches 22° C in December^57^. Table S8 (supplementary material) shows monthly precipitation data from 2016 to 2018 of several meteorological stations near the Itupararanga Reservoir from the Brazilian National Institute of Meteorology (INMET) and the Department of Water and Energy (DAAE). The reservoir was built in 1912 with the original purpose of hydroelectric power generation. Nowadays it is predominantly used as a source for drinking water for approximately one million people as well as for irrigation and leisure purposes^58^. The reservoir has maximum and mean depths of 21 and 7.8 m, respectively. The main channel has a length of about 26 km. The water residence time varies between 95 and 270 days and the reservoir has a maximum storage volume of 286 million m^3^^41,58^. The most important contributing streams are the Sorocabaçu, Sorocamirim and Una rivers next to numerous small streams (e.g. Paruru, Ressaca, and Campo Verde)^59^.

Seven locations (P1–P7) inside the Itupararanga Reservoir were sampled between December 2016 and December 2018 at depths between 0 and 15 m below the water surface (Fig. 1). Depending on the depth of the water column sampled intervals were between 1 and 5 m (cf. Tables S1–S7 in the supplementary material).

Land use in the vicinity of sampling locations P1 and P2 is dominated by grass and pasture, however P1 also receives inputs from sewage discharge. Sampling locations P3 and P4 are in the vicinity to urban areas, whereas P5 to P7 are located in parts of the catchment that are dominated by forests, agriculture and silviculture^42^.

### On-site and laboratory procedures

Temperature and pH profiles were measured at all sampling sites using a portable multiparameter probe (Horiba U-50)^42^. Samples for isotope measurements were filtered via nylon disk filters with a pore size of 0.45 μm into 40-mL amber glass vials according to standards of the U.S. Environmental Protection Agency (EPA vials). All sample vials for isotope measurements were preserved with 0.05 mL of a saturated mercuric chloride (HgCl_2_) solution in order to avoid secondary biological activities after sampling^33^.

DIC concentrations and their respective carbon isotope values (${\updelta }^{13} {\text{C}}_{{{\text{DIC}}}}$) were determined in continuous flow mode by an Aurora 1030 W TIC-TOC analyser (OI Analytical, College Station, Texas, USA) that was connected to a ThermoFisher Delta V Plus isotope ratio mass spectrometer (IRMS). The 1 − σ precision for DIC concentration measurements were better than 5‰ relative standard deviation (s.d.). The 1 − σ precision for ${\updelta }^{13} {\text{C}}_{{{\text{DIC}}}}$ analyses was determined by at least triplicate measurements of selected control samples and better than ± 0.1‰. Details of coupling the OI analyser to the IRMS and measurement techniques are described in St-Jean^60^ and van Geldern^61^.

POC samples were filtered on a glass fibre filter paper with a pore size of 0.4 μm (MN GF-5, Macherey–Nagel, Germany). Prior to sampling, filter papers were heated for 6 h at 500 °C to remove trace amounts of carbon. After filtration in the field, they were dried at 60 °C for 24 h and then pulverized in a carbon-free mortar. Pulverized samples were dried and fumigated by concentrated HCl in a desiccator for 24 h to remove possible carbonate particles. This prepared material was then weighed into tin capsules and analysed on a Costech Elemental Analyser (model ECS 4,010) linked to a ThermoFisher Delta V Plus IRMS for the analysis of the POC carbon isotope ratios (expressed as ${\updelta }^{13} {\text{C}}_{{{\text{POC}}}}$).

All isotope values are expressed in per mille (‰) against the Vienna Pee Dee Belemnite standard according to:

1 $$ \delta = \left( {\frac{{R_{sample} }}{{R_{reference} }}} \right) - 1 \times 1000 $$

where *R* is the ratio of the heavy to the light carbon isotope (i.e. ^13^C/^12^C)^62^. All isotope data were corrected for instrumental drift and linearity. All 1 − σ standard deviations for δ^13^C_POC_ at least triplicate measurements of selected samples were better than ± 0.3‰.

Chlorophyll-a (chl-a) was determined according to method of Wetzel and Likens (2000) using a 90% alkaline acetone solution for extraction of the chl-a from the 0.4 μm pore size filters. The absorbance was measured at 665 and 750 nm in a spectrophotometer for determinations of chl-a concentrations per litre. Chl-a was measured only in selected samples that were collected from surface water (1–3 m).

In order to apply the model by Rau et al.^55^ for evaluation of POC, DIC and CO_2_ relationships for algal growth the partial pressure of CO_2_ (*p*CO_2_) had to be calculated. This was done as outlined in Marx et al. (2018) by using pH and HCO_3_^-^ with the following Eq. ^63^.

2 $$ p{\text{CO}}_{2} = \frac{{{\text{HCO}}_{3}^{ - } \times H^{ + } }}{{K_{H} \times K_{1} }} $$

where HCO_3_^−^ is the concentration of bicarbonate, H^+^ is 10^−pH^, K_1_ is the temperature-dependent first dissociation constant for the dissociation of H_2_CO_3_ (mol L^−1^), and K_H_ is the Henry’s law constant in mol L^−1^ atm^−1^.

The isotope composition of CO_2_ (${\updelta }^{13} {\text{C}}_{{{\text{CO}}_{2} }}$) was calculated from DIC and the corresponding ${\updelta }^{13} {\text{C}}_{{{\text{DIC}}}}$, pH, and temperature using carbonate equilibrium constants that were linked with isotope equilibrium fractionation factors^62,64^. This led to the following equation:

3 $$ \delta^{13} C_{{{\text{CO}}_{2} }} = \frac{{\left( {\delta^{13} C_{DIC} \times \left[ {DIC} \right]} \right) - \left( {\epsilon_{P} \times \left[ {{\text{HCO}}_{3}^{ - } } \right]} \right) }}{{\left( {\left[ {p{\text{CO}}_{2} } \right] - \left[ {{\text{HCO}}_{3}^{ - } } \right]} \right)}} $$

where ${\updelta }^{13} {\text{C}}_{{{\text{CO}}2}}$ is the ${\updelta }^{13} {\text{C}}$ of the ambient CO_2(aq)_, $\epsilon_{P}$ is the temperature-dependent equilibrium isotope fractionation between HCO_3_^−^ and CO_2(aq)_ with the following equation^64–67^.

4 $$ 10^{3} ln \alpha = b \left( {\frac{{10^{3} }}{{T_{K}^{{}} }}} \right) + c $$

where *b* = 9.866, *c* = − 24.12 and T_K_ is the temperature in Kelvin^62^. The latter equation was also confirmed by other studies^66,67^. The photosynthetic fractionation factor between the isotope composition of CO_2_ and POC was calculated following the model by Rau et al.^55^:

5 $$ \epsilon_{p} = \frac{{\delta^{13} C_{{{\text{CO}}_{2} }} - \delta^{13} C_{POC} }}{{1 + \left( {\frac{{\delta^{13} C_{POC} }}{1000}} \right)}} \approx \delta^{13} C_{{{\text{CO}}_{2} }} - \delta^{13} C_{POC} $$

where $\epsilon_{p}$ is the temperature-dependent equilibrium stable isotope difference between CO_2(aq)_ and POC. Here ${\updelta }^{13} {\text{C}}_{{{\text{CO}}2\left( {{\text{aq}}} \right)}}$ was derived from Eq. (3) and ${\updelta }^{13} {\text{C}}_{{{\text{POC}}}}$ is the measured stable carbon isotope composition of the POC.

## Supplementary information

Supplementary information
