# Supplementary material for: Dominance of in situ produced particulate organic carbon in a subtropical reservoir inferred from carbon stable isotopes
Source: Sci Rep. 2020 Aug 6;10:13187. doi: 10.1038/s41598-020-69912-0 (PMC7413405; doi:10.1038/s41598-020-69912-0)

Supplementary Material

Dominance of in situ produced particulate organic carbon in a subtropical reservoir inferred from stable isotopes

Carolina de Castro Bueno[1,2]; Daniele Frascareli[2]; Erik S. J. Gontijo[2]; Robert van Geldern[1]; André H. Rosa[2]; Kurt Friese[3]; Johannes A. C. Barth[1].

[1]Friedrich-Alexander-Universität Erlangen–Nürnberg (FAU), Department of Geography and Geosciences, GeoZentrum Nordbayern, Schlossgarten 5, 91054 Erlangen, Germany

[2]São Paulo State University (UNESP), Institute of Science and Technology. Avenida Três de Março, 511. Alto da Boa Vista. 18087-180. Sorocaba – São Paulo, Brazil

[3]Helmholtz Centre for Environmental Research - UFZ, Department Lake Research, Brückstraße 3a, 39114 Magdeburg, Germany

Corresponding author: carolina.bueno@unesp.br

Table S.1. Data set for POC, DIC and carbon stable isotopes ($\delta^{13}C$) for P1.

	Depth	POC	DIC	$\delta^{13}C_{POC}$	$\delta^{13}C_{DIC}$
	**m**	***mmol/L***	***mmol/L***	***VPDB ‰***	***VPDB ‰***
DEC2016	1	0.23	0.43	-27.8	-0.8
MAR2017	1	0.15	0.57	-31.6	-5.4
MAR2017	5	0.14	0.59	-29.3	-7.3
DEC2017	1	0.22	0.51	-30.3	-2.8
DEC2017	3	0.13	0.65	-29.5	-8.8
MAR2018	1	0.09	0.41	-25.6	-3.2
MAR2018	3	0.06	0.50	-28.3	-7.3
DEC2018	1	0.10	0.50	-30.1	-7.6
DEC2018	6	0.05	0.61	-30.1	-9.3

Table S.2. Data for POC, DIC and carbon stable isotopes ($\delta^{13}C$) for P2.

	Depth	POC	DIC	$\delta^{13}C_{POC}$	$\delta^{13}C_{DIC}$
	**m**	***mmol/L***	***mmol/L***	***VPDB ‰***	***VPDB ‰***
DEC2016	1	0.19	0.41	*nd*	-1.2
MAR2017	1	0.19	0.49	-30.9	-2.1
MAR2017	6	0.15	0.53	-30.8	-4.2
DEC2017	1	0.29	0.38	-25.4	1.5
DEC2017	5	0.24	0.61	-32.2	-7.5
MAR2018	1	0.09	0.39	-25.1	-1.1
MAR2018	5	0.08	0.53	-28.4	-7.2
DEC2018	1	0.19	0.46	-28.8	-5.1
DEC2018	4	0.21	0.47	-28.7	-5.2

nd = not detected

Table S.3. Data set for POC, DIC and carbon stable isotopes ($\delta^{13}C$) for P3.

	**Depth**	**POC**	**DIC**	**$\delta^{13}C_{POC}$**	**$\delta^{13}C_{DIC}$**
	**m**	***mmol/L***	***mmol/L***	***VPDB ‰***	***VPDB ‰***
DEC2016	1	0.15	0.43	-28.2	-1.5
DEC2016	8	0.14	0.45	-28.7	-4.2
MAR2017	1	0.15	0.47	-31.6	-1.9
MAR2017	11	0.11	0.85	-37.7	-9.2
DEC2017	1	0.26	0.35	-25.2	+0.6
DEC2017	4	*nd*	0.39	*nd*	+0.3
MAR2018	1	0.12	0.44	-26.8	-0.8
MAR2018	4	0.07*	0.63	-31.4	-7.8
DEC2018	1	0.22	0.39	-27.2	-1.8
DEC2018	5	0.23	0.42	-27.0	-1.2

**unreliable analytical measure; value discarded from the analysis of the data and results presented*

nd = not detected

Table S.4. Data set for POC. DIC and carbon stable isotopes ($\delta^{13}C$) for P4.

	**Depth**	**POC**	**DIC**	**$\delta^{13}C_{POC}$**	**$\delta^{13}C_{DIC}$**
	**m**	***mmol/L***	***mmol/L***	***VPDB ‰***	***VPDB ‰***
DEC2016	1	0.15	0.43	-28.9	-1.5
MAR2017	1	0.18	*nd*	-30.4	*nd*
MAR2017	8	0.14	0.64	-33.7	-8.6
DEC2017	1	0.18	0.37	-27.2	0.3
DEC2017	8	*nd*	0.69	*nd*	-9.2
MAR2018	1	0.04	0.43	-28.9	+0.1
MAR2018	8	0.08	0.65	-33.8	-6.5
DEC2018	1	0.21	0.44	-26.8	-1.4
DEC2018	9	0.19	0.61	-29.4	-8.0

nd = not detected

Table S.5. Data set for POC, DIC and carbon stable isotopes (δ^{13}C) for P5.

	Depth	POC	DIC	$\delta^{13}C_{POC}$	$\delta^{13}C_{DIC}$
	**m**	***mmol/L***	***mmol/L***	***VPDB ‰***	***VPDB ‰***
DEC2016	1	0.15	0.39	-28.1	-0.9
DEC2016	10	0.12	0.45	-26.3	-4.3
MAR2017	1	0.13	*nd*	-31.8	*nd*
MAR2017	12	0.12	*nd*	-33.8	*nd*
DEC2017	1	*nd*	0.42	*nd*	-0.8
DEC2017	11	0.06	0.60	-32.3	-8.4
MAR2018	1	0.10*	0.36	-29.4	-3.6
MAR2018	11	0.03	0.45	-31.9	-7.2
DEC2018	1	0.15	0.43	-28.4	-2.1
DEC2018	10	0.15	0.43	-28.3	-2.3

**unreliable analytical measure; value discarded from the analysis of the data and results presented*

nd = not detected

Table S.6. Data set for POC. DIC and carbon stable isotopes (δ^{13}C) for P6.

Date	Depth	POC	DIC	$\delta^{13}C_{POC}$	$\delta^{13}C_{DIC}$
	**m**	***mmol/L***	***mmol/L***	***VPDB ‰***	***VPDB ‰***
DEC2016	1	0.14	0.39	-28.3	-1.1
DEC2016	5	0.14	0.39	-27.8	-1.0
DEC2016	10	0.16	0.43	*nd*	-2.6
MAR2017	1	0.13	0.44	-30.6	-3.0
MAR2017	15	0.13	0.48	-31.8	-4.2
DEC2017	1	0.18	0.39	-27.9	-0.3
DEC2017	15	0.14	0.76	-34.9	-10.4
MAR2018	1	0.08	0.41	-29.7	+0.1
MAR2018	15	0.07*	0.59	-35.6	-8.4
DEC2018	1	0.18	0.42	-27.6	-1.7
DEC2018	9	0.16	0.43	-27.7	-1.7

**unreliable analytical measure; value discarded from the analysis of the data and results presented*

nd = not detected

Table S.7. Data set for POC, DIC and carbon stable isotopes ($\delta^{13}C$) for P7.

Date	Depth	POC	DIC	$\delta^{13}C_{POC}$	$\delta^{13}C_{DIC}$
	**m**	***mmol/L***	***mmol/L***	***VPDB ‰***	***VPDB ‰***
DEC2016	1	0.14	0.39	-27.6	-1.2
DEC2016	5	0.14	0.42	-27.1	-1.7
DEC2016	10	0.14	0.55	-31.4	-7.6
MAR2017	1	0.12	0.41	-29.7	-2.2
MAR2017	3	0.14	0.41	-29.8	-2.1
MAR2017	7	0.12	0.44	-30.1	-3.0
MAR2017	11	0.13	0.44	-29.9	-2.9
MAR2017	15	0.12	*nd*	-32.2	*nd*
DEC2017	1	0.17	0.39	-27.3	-0.7
DEC2017	3	0.20	0.39	-27.3	-0.2
DEC2017	7	0.21	0.38	-27.6	-0.5
DEC2017	11	0.46	0.42	-27.7	-2.5
DEC2017	14	0.12	0.71	-31.7	-10.1
MAR2018	1	0.07*	0.40	-29.4	-0.2
MAR2018	3	0.03*	0.45	-29.9	-0.2
MAR2018	7	0.05*	0.43	-30.4	-3.5
MAR2018	11	0.06*	0.49	-31.7	-6.4
MAR2018	14	0.03*	0.51	-31.8	-7.7
DEC2018	1	0.16	0.41	-27.6	-1.4
DEC2018	10	0.14	0.43	-27.6	-1.7
DEC2018	14	0.07	0.53	-28.2	-7.1

**unreliable analytical measure; value discarded from the analysis of the data and results presented*

nd = not detected

Table S.8. Monthly average precipitation data (mm) obtained from meteorological stations around Itupararanga Reservoir (2016-2018).

**Year**	**Jan**	**Feb**	**Mar**	**Apr**	**May**	**Jun**	**Jul**	**Aug**	**Sep**	**Oct**	**Nov**	**Dec**	**Total**
	**Weather station: Piedade**												
**2016**	263	262	216	28	143	149	14	88	45	186	99	121	1613
**2017**	250	115	200	155	114	160	0	63	NA*	NA	NA	NA	NA
**2018**	NA	NA	NA	NA	NA	NA	NA	NA	NA	NA	NA	NA	NA
	**Weather station: Mairinque**												
**2016**	NA	NA	NA	NA	NA	NA	NA	NA	88.6	123.2	173.4	215.6	NA
**2017**	NA	112	151	162	159	145	0	93	19	146	132	135	NA
**2018**	259	101	153	NA	NA	NA	NA	NA	NA	NA	NA	NA	NA
	**Weather station: Mairinque II**												
**2016**	273	242	157	14	123	182	9	74	51	100	140	239	1604
**2017**	310	189	NA	190	187	169	0	80	17	119	NA	NA	NA
**2018**	235	75	169	29	8	NA	NA	NA	NA	NA	NA	NA	NA
	**Weather station: Sorocaba**												
**2016**	205	141	208	23	126	181	0	67	76	174	75	146	1424
**2017**	256	243	148	93	168	189	0	55	15	112	163	157	1598
**2018**	170	9	203	43	15	21	10	59	77	135	98	107	870

NA = data not available; Data from the Brazilian National Institute of Meteorology (INMET, Instituto Nacional de Metereologia - www.inmet.gov.br, accessed in January 2019) and the Department of Water and Energy (DAAE, Departamento de Águas e Energia - www.hidrologia.daee.sp.gov.br, accessed in January 2019).

Table S.9. Average wind speeds (km/h) for Sorocaba station.

**Year**	**Jan**	**Feb**	**Mrz**	**Apr**	**Mai**	**Jun**	**Jul**	**Aug**	**Sep**	**Oct**	**Nov**	**Dec**
2016	3.94	2.68	3.41	2.60	3.07	2.72	2.37	2.73	4.39	4.41	4.68	4.22
2017	2.34	3.16	2.72	-	0.87	1.70	1.77	2.26	2.70	2.79	3.06	2.79
2018	2.53	2.96	1.65	1.86	2.02	1.30	1.16	1.39	1.86	2.44	2.61	2.72

Table S.10. Sampling points and depths of samples that did not fit the fractionation model by Rau et al. (1996).

**December 2016**		**March 2017**		**December 2017**		**March 2018**		**December 2018**	
Sampling Point	*Depth (m)*	*Sampling Point*	*Depth (m)*	*Sampling Point*	*Depth (m)*	*Sampling Point*	*Depth (m)*	*Sampling Point*	*Depth (m)*
P3	8	P1	5	P1	3	P1	3	P7	14
P5	10	P6	15	P5	11	P2	5		
P7	10			P7	7	P4	8		
				P7	11	P5	14		
				P7	14	P6	15		
						P7	7		
						P7	11		
						P7	14		

Figure S.1. Entire collection of samples. (A) Theoretical (solid lines) and measured fractionation values (symbols) (ϵ), between $CO_{2(aq)}$ and phytoplanktonic POC with varying growth rates (μ), following the model by Rau et al. (1996) with (B) zoom-in of the samples. Samples allocated in the interval between the curves indicate autochthonous origin. Samples below the green solid line (μ = 2.0) do not follow the model by Rau et al. (1996) and this group indicates the samples of allochthonous origin.

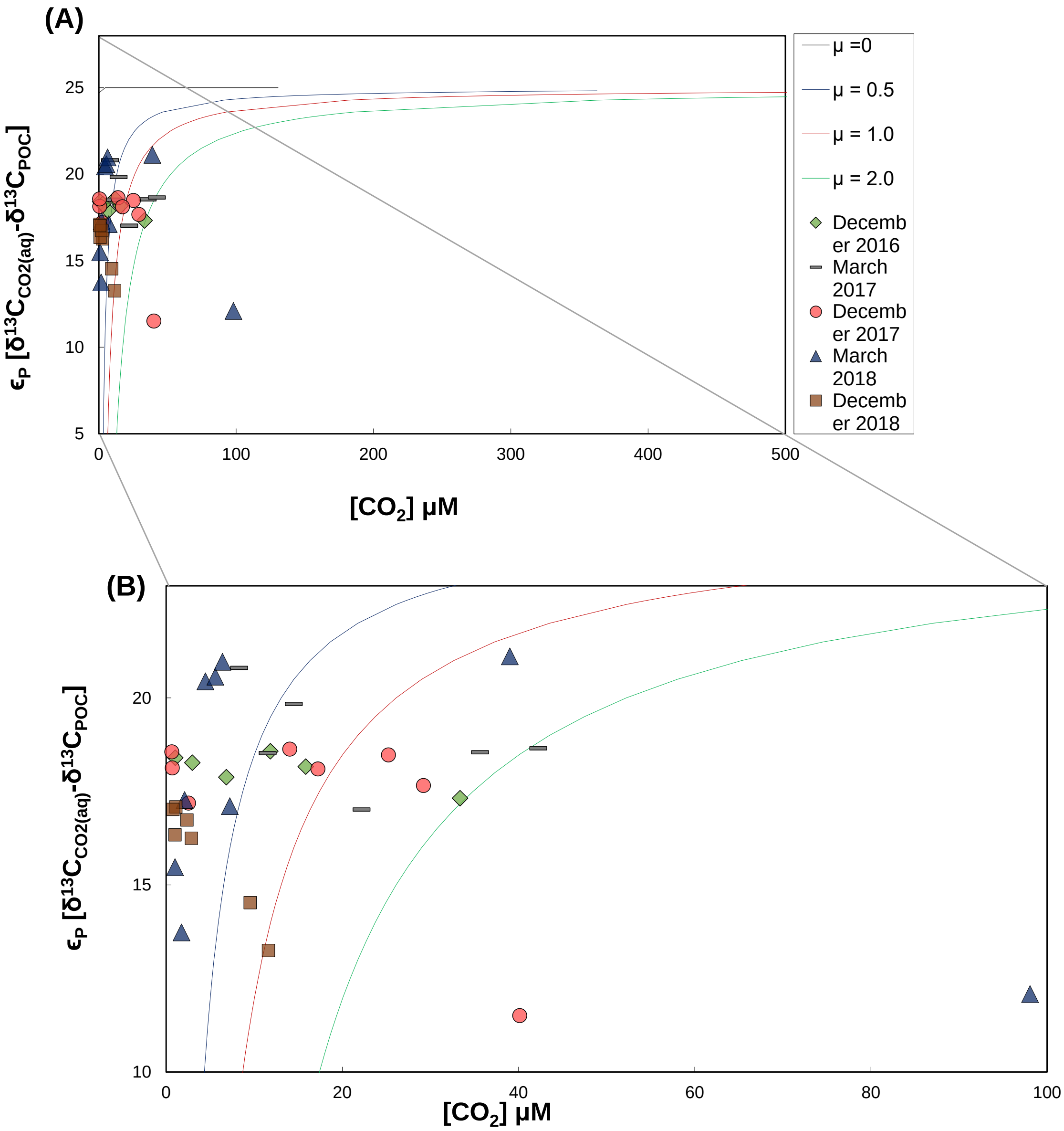

Supplement: Supplementary file 1 — Supplementary information [file 41598_2020_69912_MOESM1_ESM.pdf]
